# Does Further Education in Adulthood Improve Physical and Mental Health among Australian Women? A Longitudinal Study

**DOI:** 10.1371/journal.pone.0140334

**Published:** 2015-10-19

**Authors:** Leigh Tooth, Gita D. Mishra

**Affiliations:** School of Public Health, The University of Queensland, Brisbane, Queensland, Australia; University Of São Paulo, BRAZIL

## Abstract

**Objective:**

We analyzed whether further education in young adult and mid-life [adult educational mobility] influences physical functioning and depressive symptoms in women.

**Methods:**

14247 women born 1973–78 (younger cohort) and 13715 women born 1946–51 (mid-aged cohort) from the Australian Longitudinal Study on Women’s Health were followed for 14–16 years. Measures were the Short-Form 36 Health Survey physical functioning subscale (SF-36 PF) and Centre for Epidemiologic Studies 10-item Depression Scale (CESD-10). Linear mixed modelling, accounting for time varying covariates, assessed the influence of further education on physical functioning and depressive symptoms over time. Sensitivity analysis to assess the impact of missing data was conducted using multiple imputation.

**Results:**

Compared to younger women with a pre-existing high level of education, women gaining further education (up to age 39 years) from low levels had lower SF-36 PF scores (poorer physical functioning) (fully adjusted beta estimates (95%CIs) -1.52 (-2.59, -0.44)) while those gaining further education from middle to high levels showed equivalent SF-36 PF scores (-0.08 (-0.61, 0.44)). A similar pattern was shown for CESD-10 scores (0.78 (0.29, 1.25); -0.02 (-0.26, 0.21), respectively) where higher scores represented more depressive symptoms. For mid-age women, further education from a middle to high level resulted in equivalent SF-36 PF scores (-0.61 (-1.93,0.71)) but higher CESD-10 scores (0.49 (0.11, 0.86)), compared to highly educated women.

**Conclusion:**

Women who delay further education until they are aged between their 40s and 60s can improve or maintain their physical functioning but may have missed the critical time to minimise depressive symptomatology. Public health policy should focus on encouraging women to upgrade their educational qualifications earlier in life in order to potentially offset the negative associations between their initial lower socio-economic position class of origin and their mental health.

## Introduction

Lower levels of education are clearly linked to poorer physical and mental health in women, both cross-sectionally [1–3] and over the life course, [4–6] including in non-Western countries.[7] It is also well known that as women age their physical functioning steadily declines while their mental health generally peaks in later life before it starts to decline.[8–10] Bjelland and colleagues [4] and Ross and Mirowsky [5] have shown that education achieved in the first two decades of life appears to have a protective effect against deleterious age-related changes in physical and mental health. Although there is mixed evidence and much debate about whether the protective effects of education accumulate [2,4] or attenuate with age.[11,12]

Whether further education in later decades of life—or adult educational mobility—can delay or influence these expected changes in physical and mental health over time has been rarely studied. In one of the few published studies in this area Chandola and colleagues [13] examined the influence of further education on coronary heart disease risk. They found that women who obtained further education after the age of 23 years had a small reduction in coronary heart disease risk at age 44 years compared to women with no further education.

Adult educational mobility is rarely used as the key exposure in social mobility research. This is surprising as education is viewed as a stable, easy to administer and well answered measure of socio-economic position (SEP).[14] Most empirical research in this area has focussed on social mobility based on occupation [15–19] or income.[20,21] However, the use of occupational class is problematic as many mid-aged and older women may not have worked outside the home, and for women who do work, many retire in mid-life.[14] A downside to the use of income is the issue of non-response and missing data, and the challenge of separating the effects of individual, partner, and household income in how people report their income management.[14,22]

No research could be found that has investigated the impact of further adult education at different stages of the life course on physical functioning and mental health in women. Knowing this may help target the timing and promotion of educational interventions or programs. Thus the aim of this study was to determine whether obtaining further education as a young adult or mid-aged woman influence these women’s physical and mental health.

## Materials and Methods

### Participants

Data were from the population-based Australian Longitudinal Study on Women’s Health (ALSWH). In 1996, self-reported data on health, health service use, socio-demographic, and personal information were collected from over 41,500 women in three cohorts: those born 1973–78 (‘younger’ cohort, aged 18–23 years in 1996); those born 1946–51 (‘mid-age’ cohort, then aged 45–50 years); and those born 1921–26 (‘older’ cohort, then aged 70–75 years). The study sample was selected randomly from the Medicare Australia database, which covers all citizens and permanent residents of Australia, with oversampling of women living in rural and remote areas to ensure their continued representation over time. Since 1996, each cohort has been re-surveyed approximately every 3 years. Informed consent was obtained from all participants at each survey, with ethical clearances obtained from the University of Newcastle Human Research Ethics Committee (#H0760795) and the University of Queensland Medical Research Ethics Committee (#2004000224), in Australia. Details of recruitment and estimated initial response rates are published elsewhere.[23] Specific response cannot be determined as it is unknown whether all women who were randomly sampled received the invitation to participate. It is estimated that 41–42% of the 1973–78 cohort, 53–56% of the mid-age cohort and 37–40% of the 1921–26 cohort agreed to participate.[23] The present study includes data collected from Survey 1 to 6 from both the younger and mid-age cohorts. Retention rates are published elsewhere.[24] ALSWH data are anonymized and de-identified prior to analysis. ALSWH meta-data are deposited in the Australian Data Archive (www.ada.edu.au).

### Main exposure–further adult education

Further adult education was derived from the women’s self-reported highest own level of education asked at Surveys 1 and 6. To minimise potential biases from cohort effects, [14] cohort specific categorisations of ‘low’, ‘middle’ and ‘high’ level qualifications were defined. For the younger women ‘low’, ‘middle’ and ‘high’ represented no formal/school (up to 10 years), high school (up to 12 years)/trade/apprenticeship, and certificate/diploma/degree /higher degree qualifications, respectively. For the mid-aged women ‘low’, ‘middle’ and ‘high’ represented no formal qualifications, school/trade/apprenticeship, and certificate/diploma/degree/higher degree qualifications, respectively. For both cohorts, ‘further education’ was categorised as:

No further education

- stable low (low at Survey 1 and 6);
- stable middle (middle at Survey 1 and 6);
- stable high (high at Survey 1 and 6); or

Further education

- upwards from low (low at Survey 1 and middle/high at Survey 6)
- upwards from middle (middle at Survey 1 and high at Survey 6)

#### Outcomes

Physical functioning was measured by the Physical Functioning (PF) subscale of the well-validated Australian version of the Medical Outcomes Study Short-Form Health Survey (SF-36).[25] The responses to items within the PF subscale are summed and linearly transformed to produce dimension scores ranging from 0 to 100 (highest well-being). The PF subscale was measured at all surveys.

Depressive symptoms were measured by the Centre for Epidemiologic Studies Depression Scale–10-item version (CESD-10), which identifies depressive symptoms by self-report in nonclinical populations.[26] The CESD-10 has been shown to have good internal consistency [Cronbach’s alpha 0.80] and validity.[27] The CESD-10 is measured on a continuous scale from 0 to 30, with higher scores indicating greater depressive symptomatology. CESD-10 scores were measured from Surveys 2 to 6.

#### Socio-demographic and health behaviour covariates

Covariates with established associations with the outcomes were included to account for confounding. Unless specified these were time-varying and measured at all surveys. Socio-demographic characteristics were relationship status, area of geographical residence, [28] ability to manage on available income and a proxy of earlier life SEP (for the mid-aged women this was the ‘age they left school’, asked at Survey 1 and included as a fixed effect; for the younger women this was the ‘highest level of parental education’ (whichever was highest of their father or mother), asked at Survey 2 and included as a fixed effect). Self report height and weight were collected to enable calculation of body mass index (BMI).[29] Health behaviour characteristics were level of physical activity [30] (Surveys 2 to 6), cigarette smoking and alcohol consumption.[31] For the younger cohort the number of children the women had born by Survey 6 was included as a fixed effect.

### Statistical analysis

Directional acyclic graphs and the guidelines by Spratt [32] informed the analysis approach. Baseline characteristics were analysed descriptively. SF-36 PF and CESD-10 scores at each survey were plotted, both with and without stratification by further adult education. Linear mixed models, using the PROC MIXED command in SAS 9.3 [33] was used to analyse the effects of further education, and each group of covariates on SF-36 PF and CESD-10 scores. The analyses were conducted separately for each cohort and each outcome. For the younger cohort four stepwise models were run (further education + survey wave; + socio-demographic, SEP characteristics; + number of children the women had born; + BMI and health behaviours]. For the mid-age cohort three stepwise models were run. These were identical to those for the younger cohort with the exception that number of children born was not included. In each model for each cohort the stable high educational level (30% of women in each cohort) was the reference category. For each successive model, the beta estimates for each level of the further education exposure were compared against the previous model using a complete case approach. Sensitivity analyses for missing data were carried out using PROC MI ANALYSE in SAS. Twenty imputed datasets, including all covariates, were created and analysed with the estimates from all datasets averaged using Rubin’s rules.[34] The estimates from the imputed datasets were then compared against the estimates from the complete case approach.

## Results

Baseline descriptive statistics are shown in Table 1. At Survey 1, about half the women in both cohorts held middle level educational qualifications, with around 30% in both cohorts having higher qualifications. Further education by young women between Surveys 1 and 6 was as follows: 46.9% had no further education (5% (n = 405) were stable low; 12.3% (n = 963) were stable middle; 29.6% (n = 2314) were stable high), 6% (n = 469) had further education from low level qualifications, and 46.9% (n = 3672) had further education from middle level qualifications. For mid-aged women, 88.2% had no further education (10.8% (n = 1020) were stable low; 44.7% (n = 4206) were stable middle; 32.7% (n = 3074) were stable high), 4.8% (n = 455) had further education from low, and 6.9% (n = 647) had further education from middle level qualifications.

**Table 1:** Baseline (1996) characteristics of younger and mid-age women in the Australian Longitudinal Study on Women’s Health.

	Younger (1973–78) cohort N = 14 247	Mid-age (1946–51) cohort N = 13 715
Highest educational qualification* (%)		
Low	2427 (17.1)	2482 (18.3)
Middle	7963 (56.4)	7073 (52.1)
High	3753 (26.5)	4022 (29.6)
**Relationship status (%)**		
Married/defacto	3193 (22.5)	11311 (82.9)
Not partnered	10984 (77.5)	2336 (17.1)
**Ability to manage on income (%)**		
Not too bad/easy	6865 (48.4)	7677 (56.3)
Difficult/impossible	7330 (51.6)	5952 (43.7)
**Age left school (%)**		
14 years and under	Not applicable for cohort	1535 (11.2)
15 or 16 years		7681 (56.3)
17 years or older		4434 (32.5)
**Highest parental (mother or father) education (survey 2)** ^**†**^ **(%)**		
Up to gr 10	2362 (24.9)	Not applicable for cohort
11 or 12 years	1174 (12.4)	
Trade/certificate/diploma	2705 (28.6)	
Degree/Higher Degree	2242 (23.7)	
Don’t Know/Not Applicable	971 (10.3)	
**Area of residence (%)**		
Major city	7375 (51.8)	5000 (36.5)
Inner regional	4307 (30.3)	5214 (38.0)
Outer regional/remote	2555 (17.9)	3498 (25.5)
**Number of children by Survey 6** ^**‡**^ **(%)**		
None	2116 (26.4)	Not applicable for cohort
One	1265 (15.8)	
Two	2883 (35.9)	
Three or more	1746 (21.8)	
**Body mass index (%)**		
Acceptable/underweight (BMI ≤25)	9606 (78.4)	6923 (52.5)
Overweight (25<BMI<30)	1875 (15.3)	3804 (28.9)
Obese (BMI ≥30)	772 (6.3)	2452 (18.6)
**Physical activity (%) at survey 2** ^**†**^		
Nil/sedentary/low	4240 (44.9)	5498 (49.0)
Moderate/high	5196 (55.1)	5728 (51.0)
**Smoking status (%)**		
Current	4421 (32.4)	2431 (18.3)
Ex-smoker	2085 (15.3)	3776 (28.5)
Never smoker	7123 (52.3)	7050 (53.2)
**Alcohol consumption (%)**		
Rarely/non drinker	6109 (43.4)	6335 (46.6)
Low/high risk drinker	7979 (56.6)	7249 (53.4)
**SF-36 physical functioning subscale mean (SD)**	90.1 (15.5)	85.4 (18.5)
**CESD-10 subscale at survey 2 mean (SD)** ^**†**^	7.61 (5.5)	6.47 (5.6)

* For 1973–78 cohort Low = no formal education/school to grade 10, Middle = high school/trade/apprenticeship, high = certificate/diploma/degree/higher degree; for 1946–51 cohort Low = no formal schooling, Middle = school to grade 10/high school/ trade/apprenticeship, high = certificate/diploma/degree/higher degree.

^†^ Highest parental education (for younger cohort), physical activity and CESD-10 first measured at Survey 2, conducted in 1999 for younger cohort (n = 9688), and in 2000 for mid-age cohort (n = 11226).

^‡^ n = 8010

SD = standard deviation

**Fig 1:**
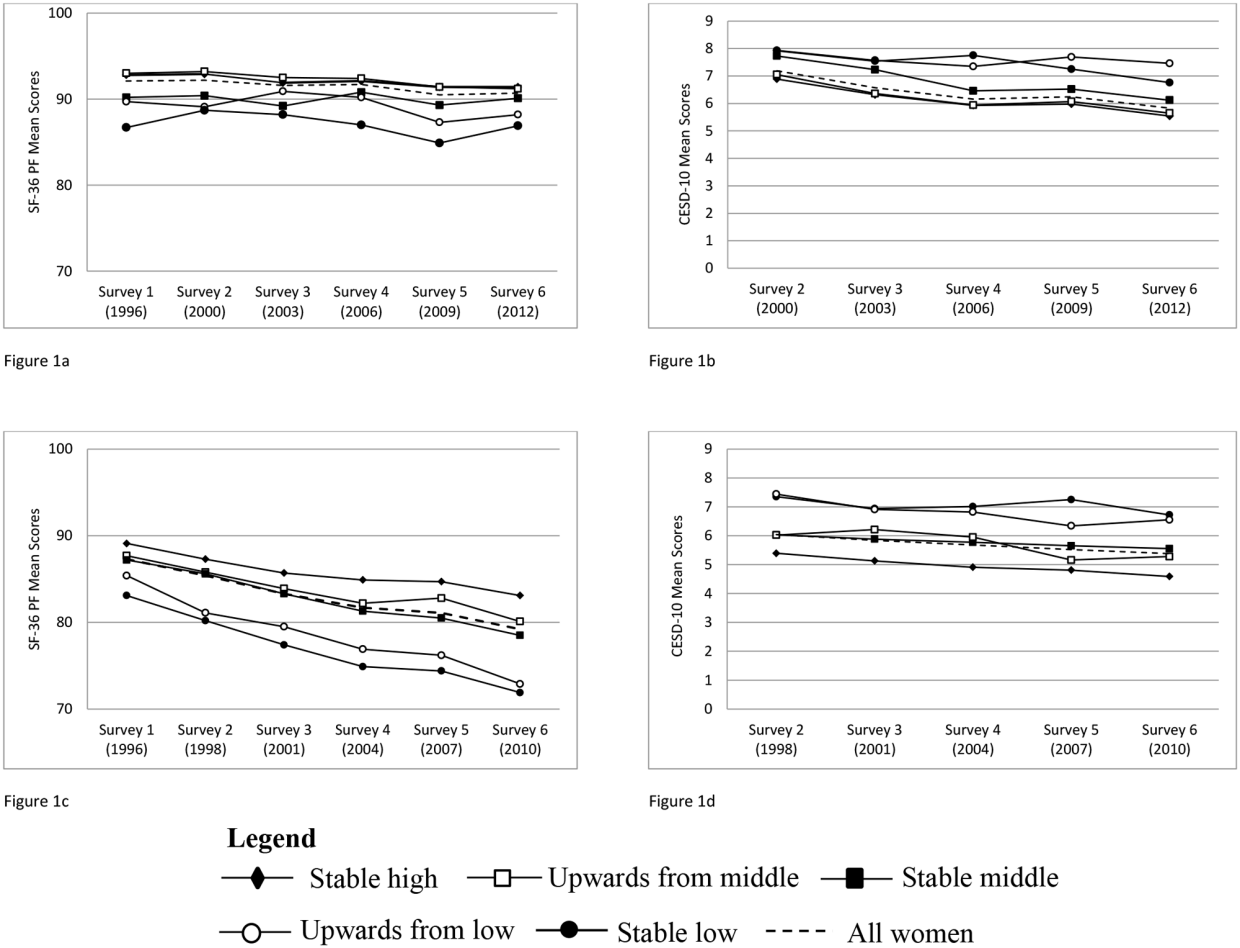
Mean scores across surveys, and by levels of educational mobility, of the main outcomes for women in both ALSWH cohorts: 1a—SF-36 PF scores at surveys 1 to 6 for 1973–78 cohort; 1b—CES-D scores at surveys 2 to 6 for 1973–78 cohort; 1c—SF-36 PF scores at surveys 1 to 6 for 1946–51 cohort; 1d—CES-D scores at surveys 2 to 6 for 1946–51 cohort.

Fig 1 shows the unadjusted mean SF-36 PF and CESD-10 scores at each survey for both cohorts. Mean SF-36 PF scores for all the younger women ranged between 92.1 (SE 0.17) at Survey 1 to 90.7 (SE 0.21) at Survey 6 (Fig 1a), while CESD-10 scores slightly decreased (indicating less depressive symptoms) from 7.2 (SE 0.08) at Survey 2 to 5.8 (SE 0.07) at Survey 6 (Fig 1b). Mean SF-36 PF scores for all mid-aged women steadily declined (indicating worsening physical functioning) from 87.4 (SE 0.18) to 79.2 (SE 0.23) (Fig 1c). Mean CESD-10 scores showed a slight decrease from 6.0 (SE 0.06) from Survey 2 to 5.4 (SE 0.06) at Survey 6 (Fig 1d).

When stratified by further education, Fig 1a and 1c show the women in the stable low education group in both cohorts had the lowest mean SF-36 PF scores. Fig 1a also shows greater fluctuation in SF-36 PF scores over time for the women in the upwards from low education group. Women in the stable low and upwards from low education groups in both cohorts showed steadily higher mean CESD-10 scores, representing more depressive symptoms, and greater fluctuation in scores over time (Fig 1b and 1d).

### Impact of further education on physical functioning

In both cohorts education was consistently significantly related to SF-36 PF scores after controlling for covariates (Tables 2 and 3, all models). Women with a stable low or middle qualification or those upwardly mobile from low had significantly lower SF-36 PF scores compared to those with a stable high education. Women upwardly mobile from middle to high had equivalent scores to those with stable high education. A clear gradient in beta estimates for each level of education was apparent (e.g, for the younger and mid-aged women the beta estimates for the upwards from low and upwards from middle education groups, compared to the stable high group, were -1.52 and -0.08, and -3.77 and -0.61, respectively). When expressed as effect sizes, Cohen’s d [35] of the Survey 6 differences in means between the stable low, upwards from low, stable middle and upwards from middle groups compared to the stable high group were -0.27, -0.19, -0.02 and -0.09 respectively for the younger women (small differences), and -0.51, -0.48, -0.23 and -0.16 respectively for the mid-aged women (medium to small differences).

**Table 2:** Effect of Further Adult Education on SF-36 Physical Functioning and CESD-10 Scores in Young Women: Regression Estimates and 95% Confidence Intervals (CIs).

	Model A		Model B		Model C		Model D		Model E (Fully adjusted multiple imputation model)	
	β	95 CIs	β	95 CIs	β	95 CIs	β	95 CIs	β	95 CIs
**Outcome: SF-36 PF Scores** ^†^										
*Further education*										
Stable low	-5.08***	-6.30, -3.86	-4.63***	-5.88, -3.37	-4.69***	-5.94, -3.44	-3.43***	-4.64, -2.23	-3.47***	-4.54, -2.39
Upwards from low	-2.86***	-4.01, -1.72	-2.45***	-3.59, -1.30	-2.39***	-3.53, -1.25	-1.52**	-2.59, -0.44	-1.38**	-2.33, -0.43
Stable middle	-2.13***	-2.95, -1.31	-1.86***	-2.69, -1.03	-1.91***	-2.73, -1.08	-1.04**	-1.82, -0.26	-0.85*	-1.55, -0.14
Upwards from middle	0.19	-0.37, 0.76	0.12	-0.45, 0.68	0.24	-0.33, 0.80	-0.08	-0.61, 0.44	0.06	-0.42, 0.54
Stable high *(ref)*	0		0		0		0		0	
**Outcome: CESD-10 Scores** ^‡^										
*Further education*										
Stable low	1.28***	0.74, 1.81	0.75**	0.23, 1.28	0.82**	0.30, 1.34	0.58*	0.05, 1.11	0.61*	0.14, 1.08
Upwards from low	1.48***	0.99, 1.98	1.02***	0.54, 1.49	0.99***	0.52, 1.48	0.78**	0.29, 1.25	0.59**	0.17, 1.01
Stable middle	0.68**	0.33, 1.04	0.53**	0.19, 0.88	0.57**	0.22, 0.91	0.38*	0.03, 0.72	0.36*	0.05, 0.67
Upwards from middle	0.08	-0.16, 0.33	0.02	-0.22, 0.25	-0.07	-0.30, 0.17	-0.02	-0.26, 0.21	-0.01	-0.22, 0.20
Stable high *(ref)*	0		0		0		0		0	

Model A to D complete case analysis,

^†^N = 5033,

^‡^N = 5032;

Abbreviations: β (95 CIs)–multiple regression estimate (SF-36 Physical Function Subscale, CSED-10 Scores) and 95% confidence Intervals.

*P ≤ 0.05;

**P ≤ 0.01;

***P ≤ 0.001;

Model A = adult educational mobility; Model B = model A + other SES (area of residence + manage on income + marital status + parental education); Model C = model B + number of children; Model D = Model C + lifestyle factors (body mass index, alcohol, smoking, physical activity); Model E = Fully adjusted multiple imputation model using 20 imputations.

**Table 3:** Effect of Further Adult Education on SF-36 Physical Functioning and CESD-10 Scores in Mid-aged Women: Regression Estimates and 95% Confidence Intervals (CIs).

	Model A		Model B		Model C		Model E (Fully adjusted multiple imputation model)	
	β	95 CIs	β	95 CIs	β	95 CIs	β	95 CIs
**Outcome: SF-36 PF Scores** ^†^								
*Further education*								
Stable low	-8.86**	-10.0, -7.70	-6.19**	-7.54, -4.85	-4.81**	-6.09, -3.52	-5.39**	-6.58, -4.19
Upwards from low	-7.18**	-8.81, -5.56	-4.81**	-6.52, -3.11	-3.77**	-5.39, -2.14	-3.76**	-5.26, -2.27
Stable middle	-3.07**	-3.81, -2.32	-2.04**	-2.87, -1.21	-1.39**	-2.18, -0.61	-1.70**	-2.45, -0.96
Upwards from middle	-2.04**	-3.43, -0.65	-1.20	-2.59, 0.19	-0.61	-1.93, 0.71	-0.81	-2.03, 0.42
Stable high *(ref)*	0		0		0		0	
**Outcome: CESD-10 Scores** ^‡^								
*Further education*								
Stable low	2.09**	1.78, 2.42	1.61**	1.24, 1.98	1.38**	1.02, 1.75	1.39**	1.05, 1.73
Upwards from low	1.87**	1.42, 2.32	1.43**	0.97, 1.89	1.19**	0.74, 1.66	1.31**	0.88, 1.73
Stable middle	0.82**	0.62, 1.03	0.67**	0.44, 0.89	0.57**	0.34, 0.79	0.61**	0.39, 0.82
Upwards from middle	0.78**	0.39, 1.16	0.55*	0.17, 0.93	0.49*	0.11, 0.86	0.55*	0.21, 0.90
Stable high *(ref)*	0		0		0		0	

Model A to C complete case analysis,

^†^N = 7944,

^‡^N = 7934;

Abbreviations: β (95 CIs)–multiple regression estimate (SF-36 Physical Function Subscale, CSED-10 Scores) and 95% confidence Intervals.

*P ≤ 0.01;

**P ≤ 0.001;

Model A = adult educational mobility; Model B = model A + other SES (area of residence + manage on income + marital status + age left school); Model C = model B + lifestyle factors (body mass index, alcohol, smoking, physical activity); Model D = Fully adjusted multiple imputation model using 20 imputations.

### Impact of further education on depressive symptoms

In both cohorts, a clear significant gradient in CESD-10 scores by level of education and further education was shown (Tables 2 and 3). Younger women who were upwardly mobile from middle to high had equivalent scores to those with stable high education. For the mid-aged women, even if they obtained further education from middle to high, they still had significantly higher CESD-10 scores (0.49 units higher), representing more depressive symptoms, compared to women who were stable high. Cohen’s d of the Survey 6 differences in means between the stable low, upwards from low, stable middle and upwards from middle groups compared to the stable high group were 0.24, 0.34, 0.12 and 0.02 respectively for the younger women (small differences), and 0.42, 0.37, 0.19 and 0.14 respectively for the mid-aged women (medium to small differences).

All multiple imputation sensitivity analyses produced largely equivalent regression estimates to the complete case analyses (final columns in Tables 2 and 3).

## Discussion

Our study offers insights into how obtaining further education at different stages of the adult life course influences women’s physical and mental health. The first insight is that obtaining further education up to age 39 for younger women or at any time between the ages of 45 and 64 years for mid-aged women was associated with optimal physical functioning. That is, this high level of education did not have to be obtained earlier for it to have an impact. This result was found only for women who upgraded from a middle level education to a high level. Women in both age cohorts who obtained further education from a low level showed better physical functioning than those who remained at a low level but poorer physical functioning compared with those with a stable middle or high education.

The second insight was that there appeared to be a similar protective effect of obtaining further education on depressive symptoms for younger women. For the younger women, further education to a high level that was achieved up to the age of 39 was associated with less depressive symptoms. For mid-aged women, even if they obtained the highest further education by the age of 64 years, they remained with more depressive symptoms than women who had obtained their highest education before the age of 45 (CESD-10 estimate 0.49 (95%CIs 0.11, 0.86) units higher). Thus we may speculate that although the highest level of educational attainment was associated with minimising depressive symptoms for both cohorts, and younger women can gain this benefit if they increase their educational qualifications in their 20s and 30s, mid-aged women may not attain this benefit, even if they upgrade their qualifications in their 50s and 60s.

These first insights may fit a social causation perspective that low education increases the risk of poorer physical functioning and depressive symptoms. Educationally mobile younger and mid-aged women showed SF-36 PF and CESD-10 scores in between their class of origin and destination. Young women with the greatest further education (middle to high) reached their class of destination for both SF-36 PF and CSED-10. However, for depressive symptoms no matter how upwardly mobile the mid-age women were, they never achieved equivalent scores as the destination class, although the difference between the two highest classes was small. This cohort difference may reflect that factors that mediate or affect the relationship between educational attainment and depressive symptoms differ by life stage/age. Bjelland and colleagues [4] showed education to have a stronger effect on mental health in later life (over 64 years of age) compared to earlier life (less than 44 years), and suggested the association between education and mental/cognitive resources such as resilience may be stronger in younger adults, thus possibly offering greater protection against mental health symptoms. Lorant and others [2] found that low SEP is more strongly associated with persistence of depression than incidence. Thus people who can upgrade their educational qualifications earlier in life may be able to offset the negative associations between their initial lower SEP class of origin and their mental health.

To aid interpretation of the physical functioning results, we can compare the SF-36 PF beta estimates for the younger and mid-aged women with unstandardized normative SF-36 PF scores for Australian women in similar age groupings in 1995.[36] Overall, the mean SF-36 PF scores for women in the current study showed they had equivalent or better physical functioning than average Australian women. However, the beta estimates showed that women in the stable low and upwards from low groups had SF-36 PF scores that were between 7 to 9 units lower, and 2 to 3 units lower for women in the stable middle or upwards from middle groups. To contextualise these results, a difference of 5 units in SF-36 PF scores is equivalent to differences observed in the Australian normative scores for people with chronic conditions like hypertension, diabetes, asthma and injury [36] compared to women without those conditions.

Cohort differences were apparent in this study and must be considered a potential limitation. Almost 53% of the younger women added educational qualifications compared with 12% of mid-aged women (due to improved education participation rates in Australia). We did create the educational mobility categories to reflect cohort differences.[14,37] We acknowledge that years of education completed may not reflect the quality of the education received or whether true educational accumulation/mobility has occurred [13,14] although we attempted to capture earlier life SEP. We were also unable to separately model mobile from low to middle or low to high due to small numbers. Other limitations included not knowing the childhood health, of the women, including physical functioning and depressive symptoms, which may have influenced later life health outcomes [38] and educational attainment (reverse causation). We only focussed on education. There were other SES measures available including occupation however we had the problem of interpreting meaningful changes in occupational status particularly in mid-aged women with high levels of retirement. Further there were high missing values with income measures.

The study was unique in that repeated measures over 14 to 16 years from the same women over two periods in the life span were able to be analysed. Other strengths were that the ALSWH is a nationally representative sample with good retention over time. These results are generalizable to similarly aged women in countries with similar education systems. We used fully adjusted models and the results were confirmed by multiple imputation.

### Conclusion

Declines in physical and mental health in women as they age can be moderated by further educational attainment but life stage is important–going back to school as a mid-aged woman when starting off a low educational level may not benefit depressive symptoms as much as physical functioning. Public health policy should focus on encouraging women to upgrade their educational qualifications earlier in life in order to potentially offset the negative associations between their initial lower socio-economic position class of origin and their mental health.
